# A closer look at the increase in suicide rates in South Korea from 1986–2005

**DOI:** 10.1186/1471-2458-9-72

**Published:** 2009-02-27

**Authors:** Jin-Won Kwon, Heeran Chun, Sung-il Cho

**Affiliations:** 1 Department of Epidemiology, School of Public Health and Institute of Health and Environment, Seoul National University, Seoul, Korea; 2 Department of Pharmacy Practice and Administration, School of Pharmacy, Rutgers University, New Jersey, USA

## Abstract

**Background:**

Suicide rates have recently been decreasing on average among OECD countries, but increasing trends have been detected in South Korea, particularly since the 1997 economic crisis. There have been no detailed analyses about the changes of the suicide rates over time periods in Korea. We examined trends in both absolute and proportional suicide rates over the time period of economic development, crisis, and recovery (1986 – 2005) as well as in birth cohorts from 1924 to 1978.

**Methods:**

We used data on total mortality and suicide rates from 1986 to 2005 published online by the Korean National Statistical Office (NSO) and extracted data for individuals under 80 years old. The analyses of the trends for 1) the sex-age-specific total mortality rate, 2) the sex-age-specific suicide rate, and 3) the sex-age-specific proportional suicide rate in 1986–2005 were conducted. To demonstrate the birth cohort effect on the proportional suicide rate, the synthetic birth cohort from 1924 to 1978 from the successive cross-sectional data was constructed.

**Results:**

Age standardized suicide rates in South Korea increased by 98% in men (from 15.3 to 30.3 per 100,000) and by 124% in women (from 5.8 to 13.0 per 100,000). In both genders, the proportional increase in suicide rates was more prominent among the younger group aged under 45, despite the absolute increase being attributed to the older group. There were distinct cohort effects underlying increasing suicide rates particularly among younger age groups.

**Conclusion:**

Increasing suicide rates in Korea was composed of a greater absolute increase in the older group and a greater proportional increase in the younger group.

## Background

Suicide is a dramatic example of individual behaviour influenced by social integration or regulation, as originally noted by Durkheim [1]. Therefore, not only individual factors but also socioeconomic changes should be considered to explain suicide patterns in a society. According to World Health Organisation (WHO) statistics from 1965 to 1999, suicide rates had a variation and showed mixed trends between countries and age-groups. While total suicide mortality rates in all ages have been decreasing or in a steady status in most developed countries after 1990s, it has been increasing in some countries especially which have suffered huge economic turmoil such as Russia. There are some reports on suicide rates by age group. In a few countries including New Zealand and Australia, there were rising trends in young people [2,3]. In such countries as Japan and Hongkong, suicide rates significantly increased with increasing age [4]. The rapid increase of numbers of elderly people led to a higher elderly dependency ratio and insufficient support in society, possibly resulting in the increase in the suicide rate among the old people [5]. Although the trends vary by countries and reveal marked age and gender differences, suicide remains a growing social and public health issue in many regions worldwide, necessitating detailed analysis of its patterns of occurrence.

Proportional mortality has been used in the analysis of age-specific patterns for monitoring disease trends [6]. The measure for suicide has received little attention in the literature, despite the relative importance of suicide in the decrease in total age-specific mortality in most developed countries. Since there could be a significant age difference in suicide epidemiology according to cultural and socio-structural background, proportional mortality from suicide can be a useful measure for in-depth analysis of the patterns of suicide.

In 2004, among 30 OECD countries, South Korea ranked second after Japan, with a suicide rate of 23.8 per 100,000, with a further increase in 2005. As in other countries, age differences are notable in suicide patterns in South Korea, where the elderly over 65 historically and currently showed the highest suicide rate. Suicide rates were relatively higher among females than among their male counterparts in the OECD gender-specific comparison: Korea's females ranked first at a rate of 15.0, while males ranked third at 32.5 per 100,000 [7,8].

**Figure 1:**
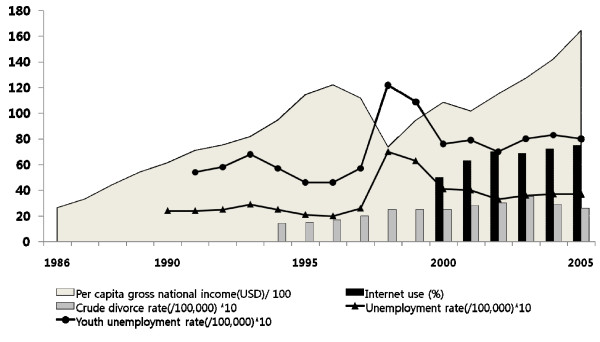
**Per capita gross national income, unemployment rates, crude divorce rates, and internet use between 1986 and 2005 in South Korea**.

Thus, South Korea could provide a unique setting to enhance our understanding of the impact of rapid industrialisation, urbanisation, or demographic changes (*i.e*., aging, unemployment, divorce rate) on age-specific suicide patterns. The country is well known for its unprecedented economic growth since 1970's, with a 60-fold increase in the per capita gross national income to US$16,400 in 2005 [9]. However, there was a brief downturn due to an economic crisis in late 1997, which left various detrimental impacts on society and accordingly influenced the suicide rate among Koreans. Per capita gross national income plummeted from US$11,200 in 1997 to US$7,400 in 1998. The Korean economy has subsequently recovered and the social-economic situation including unemployment rates [10], divorce rates [11], and internet usage [12] showed very rapid fluctuations (Figure. 1). To assess whether suicide rates changed differently by age groups in these social contexts, we examined trends in both absolute and proportional suicide rates over the time period from 1986 to 2005, specifically in the birth cohorts from 1924 to 1978.

## Methods

We used data on total mortality and suicide rates from 1986 to 2005 published online by the Korean National Statistical Office (NSO) based on the certification of death and extracted data for individuals under 80 years old [13]. These data provide the total mortality and cause-specific mortality rates by five-year intervals in age group and gender. The mortality rate was expressed as the number of deaths per 100,000 using the estimated mean population of the five-year interval age group in each year. The cause of death on the certificates was classified based on the 9^th ^and 10^th ^revision of the International Classification of Disease (ICD-9 and ICD-10). For the suicide rate, the intentional self harm classification (E950–959 or X60–X84) of ICD-9 or 10 was used. The suicide and total mortality rate was age-standardized using the standard population in 2005. The proportional suicide rate was expressed as the percentage of the number of suicide deaths in an age group divided by the total number of deaths from all causes in that age group.

Using these data, analyses of the trends for 1) the sex-age-specific total mortality rate, 2) the sex-age-specific suicide rate, and 3) the sex-age-specific proportional suicide rate in 1986–1990, 1991–1995, 1996–2000, and 2001–2005 were conducted. To demonstrate the birth cohort effect on the proportional suicide rate, we constructed the synthetic birth cohort from 1924 to 1978 from the successive cross-sectional data. The aggregate birth cohort was restructured into 11 groups, each spanning 5 years, following the standard approach to cohort analysis (Table 1). For the description and figuring of trends, we used SAS 9.1 statistical package and microsoft excel program.

**Table 1:** Total mortality and suicide rates by time period (1986–2005) and birth cohort (1924–1978)

		Year (1986–90)			Year(1991–95)			Year(1996–00)			Year(2001–05)		
Gender	Age (years)	Suicide Rates**	Total Mortality Rates**	Birth cohort***	Suicide Rates**	Total Mortality Rates**	Birth cohort***	Suicide Rates**	Total Mortality Rates**	Birth cohort***	Suicide Rates**	Total Mortality Rates**	Birth cohort***

	5 – 9 *	0.0	77.9		0.1	53.2		0.2	34.3		0.1	23.4	
Men	10 – 14	**1.1**	**56.2**	**1974–78**	1.3	41.1		1.5	28.3		0.9	18.4	
	15 – 19	**9.5**	**127.6**	**1969–73**	**9.1**	**113.4**	**1974–78**	9.3	83.6		7.6	46.7	
	20 – 24	**16.5**	**155.0**	**1964–68**	**13.3**	**125.6**	**1969–73**	**16.5**	**104.2**	**1974–78**	14.0	64.6	
	25 – 29	**17.3**	**204.8**	**1959–63**	**14.7**	**159.7**	**1964–68**	**19.1**	**122.2**	**1969–73**	**19.9**	**81.5**	**1974–78**
	30 – 34	**15.5**	**256.4**	**1954–58**	**15.2**	**211.8**	**1959–63**	**21.9**	**158.1**	**1964–68**	**23.9**	**109.1**	**1969–73**
	35 – 39	**15.5**	**379.5**	**1949–53**	**16.4**	**313.0**	**1954–58**	**25.6**	**246.2**	**1959–63**	**28.7**	**178.4**	**1964–68**
	40 – 44	**16.4**	**564.2**	**1944–48**	**16.4**	**485.0**	**1949–53**	**29.2**	**392.2**	**1954–58**	**34.2**	**300.5**	**1959–63**
	45 – 49	**19.9**	**932.0**	**1939–43**	**18.7**	**718.5**	**1944–48**	**33.8**	**603.6**	**1949–53**	**40.9**	**477.3**	**1954–58**
	50 – 54	**19.5**	**1270.0**	**1934–38**	**22.0**	**1095.6**	**1939–43**	**35.4**	**865.0**	**1944–48**	**47.3**	**711.0**	**1949–53**
	55 – 59	**19.8**	**1773.3**	**1929–33**	**21.4**	**1570.9**	**1934–38**	**37.8**	**1342.4**	**1939–43**	**53.2**	**1026.3**	**1944–48**
	60 – 64	**23.8**	**2752.5**	**1924–28**	**23.3**	**2351.8**	**1929–33**	**42.3**	**2020.0**	**1934–38**	**65.2**	**1619.4**	**1939–43**
	65 – 69	26.6	4281.6		**28.5**	**3644.1**	**1924–28**	**45.9**	**3089.1**	**1929–33**	**80.5**	**2564.6**	**1934–38**
	70 – 74	32.5	6583.6		34.0	5745.5		**54.6**	**5011.1**	**1924–28**	**95.9**	**4176.0**	**1929–33**
	75 – 79	37.4	10530.0		35.1	8675.7		69.6	7939.5		**127.0**	**6917.7**	**1924–28**

	Age standardized	15.3	961.4		15.0	811.8		23.7	686.5		30.3	551.9	

Women	5 – 9*	0.0	61.7		0.1	37.6		0.2	24.2		0.0	16.9	
	10 – 14	**0.8**	**43.5**	**1974–78**	1.2	27.9		1.8	19.1		1.2	13.8	
	15 – 19	**4.8**	**61.9**	**1969–73**	**4.8**	**46.0**	**1974–78**	7.2	38.8		5.8	24.0	
	20 – 24	**7.4**	**79.9**	**1964–68**	**7.2**	**57.8**	**1969–73**	**9.4**	**45.7**	**1974–78**	10.9	34.6	
	25 – 29	**7.6**	**91.0**	**1959–63**	**7.5**	**66.1**	**1964–68**	**10.2**	**52.0**	**1969–73**	**11.3**	**41.0**	**1974–78**
	30 – 34	**6.2**	**106.6**	**1954–58**	**7.4**	**84.4**	**1959–63**	**9.7**	**67.8**	**1964–68**	**11.8**	**53.6**	**1969–73**
	35 – 39	**6.5**	**151.8**	**1949–53**	**7.1**	**112.3**	**1954–58**	**10.5**	**95.6**	**1959–63**	**13.8**	**76.2**	**1964–68**
	40 – 44	**5.5**	**221.9**	**1944–48**	**6.5**	**163.8**	**1949–53**	**9.5**	**134.9**	**1954–58**	**12.9**	**110.8**	**1959–63**
	45 – 49	**5.5**	**344.6**	**1939–43**	**6.9**	**261.1**	**1944–48**	**9.5**	**199.9**	**1949–53**	**13.5**	**161.7**	**1954–58**
	50 – 54	**5.3**	**493.6**	**1934–38**	**6.1**	**394.6**	**1939–43**	**9.1**	**302.2**	**1944–48**	**13.7**	**240.0**	**1949–53**
	55 – 59	**5.9**	**729.2**	**1929–33**	**6.1**	**591.3**	**1934–38**	**10.1**	**466.4**	**1939–43**	**14.4**	**362.5**	**1944–48**
	60 – 64	**6.8**	**1129.4**	**1924–28**	**8.0**	**966.0**	**1929–33**	**11.8**	**769.4**	**1934–38**	**17.7**	**597.2**	**1939–43**
	65 – 69	9.6	1938.6		**10.0**	**1658.7**	**1924–28**	**14.1**	**1372.3**	**1929–33**	**24.9**	**1068.2**	**1934–38**
	70 – 74	10.9	3386.0		12.7	2986.9		**20.4**	**2578.3**	**1924–28**	**37.4**	**2048.2**	**1929–33**
	75 – 79	13.0	5862.6		16.7	5220.7		26.2	4720.0		**54.7**	**4040.3**	**1924–28**

	Age standardized	5.8	549.8		6.5	462.8		9.3	388.9		13.0	313.3	

* Suicide rates were not reported in age group of 0–4 years old

** Suicide rates and total mortality rates (per 100,000)

** *Birth cohort = Year (median year during the periods) – Age

## Results

**Figure 2:**
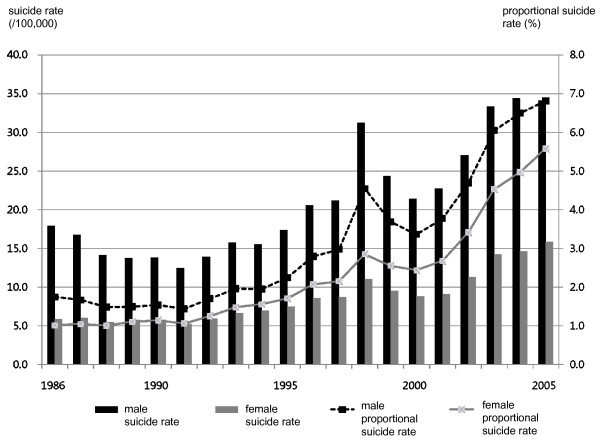
**Suicide rates and proportional suicide rates between 1986 and 2005 in South Korea**.

The age adjusted suicide rate and proportional suicide rate by each year from 1986 to 2005 were presented in Figure 2. The economic crisis before and after 1997/98 appeared to affect sudden increase of suicide mortality for the short term and the mortality showed the continuous increase till 2005.

Careful comparison of Figure 1 and 2 suggests conflicting patterns of correspondence between macro-social indicators and suicide rates. For example, per capita gross national income trends in Figure 1 had two distinct components: a long-term increase and a short term drop around 1998, which were in opposite directions. In contrast, suicide rates in Figure 2 showed the same increasing patterns corresponding to the two opposite changes in per capita gross national income.

The total mortality and suicide rate by a five-year period and birth cohort were presented in Table 1. The age-standardized total mortality rate decreased by 42% in men (from 961 to 552 per 100,000) and by 43% in women (from 550 to 313 per 100,000) between 1986 and 2005. The age-standardized suicide rate increased by 98% in men (from 15.3 to 30.3 per 100,000) and by 124% in women (from 5.8 to 13.0 per 100,000) between 1986 and 2005. The proportional suicide rate increased by 244% in men (from 1.6 to 5.5%) and by 282% in women (from 1.1 to 4.2%) between 1986 and 2005.

**Figure 3:**
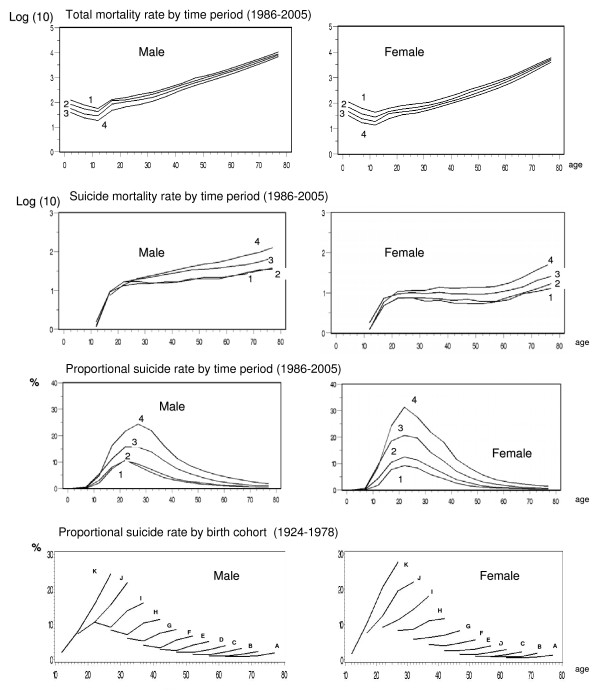
**Sex-age specific total mortality rates, suicide rates and proportional suicide rates from 1986 to 2005 and proportional suicide mortality by cohort from 1924 to 1978**. Time period: 1 = 1986–1990 2 = 1991–1995 3 = 1996–2000 4 = 2001–2005. Birth cohort: A: 1924–1928, B: 1929–1933, C: 1934–1938, D: 1939–1943, E: 1944–1948, F: 1949–1953, G: 1954–1958, H: 1959–1963, I: 1964–1968, J: 1969–1973, K: 1974–1978.

The trends on sex-age specific total mortality rate, suicide rates, and proportional suicide rates between 1986 and 2005 and the proportional suicide mortality by cohort from 1924–1978 are presented in Figure 3. A clear contrast was apparent between the decreasing total mortality and the increasing suicide mortality over time, across all ages. This led to drastic increase of proportional mortality from suicide. There was also a remarkable difference in the patterns between the age groups. The absolute suicide rate increased significantly among elderly people. The proportional suicide rate showed a steeper increase among the younger group under 45. In particular, younger women showed the steepest increase in the later period. Finally, there were distinct cohort effects among all age groups in the proportional mortality from suicide. The younger birth cohort (those born later) showed a higher proportional suicide rate compared to older cohorts.

## Discussion

Our results showed that the suicide rate in South Korea increased, while total mortality decreased, from 1986 to 2005. We observed a trend in age- and gender-specific proportional mortality rates for suicide. In both genders, the proportional increase in suicide rates was more prominent among the younger group aged under 45, despite the absolute increase being attributed to the older group.

### Decrease in total mortality

There are several explanations for the decrease in total mortality in South Korea in recent decades. Economic development, improved hygiene, and advances in medicine most likely all contributed to the decrease in total mortality and increase in longevity in the country. Also, the establishment of a national social welfare system has promoted advances in health and longevity. The Korean government initiated the National Health Insurance system in 1977 (originally starting with civil servants and employees) and has expanded to cover the whole population since 1989 [14]. Medical progress and easier access to health services have helped to decrease the incidence of cancer and cerebro-vascular diseases [15], which are the main causes of death in Korea. There was also a marked decrease in mortality due to traffic accidents, while the rate of suicidal deaths increased significantly.

### Proportional suicide mortality according to age cohort

The proportional mortality rate could be very useful in suicide epidemiology for the detection of age-specific patterns and for setting a primary target to lower the level of total mortality in a population. From our data, substantial cohort effects on the proportional suicide rate were observed (Figure. 3). Among the decreasing total mortality rate of Koreans over the last decade, the younger birth cohort (persons born later) exhibited an additional and steeper increase in the proportion of suicide to total mortality compared to the older birth cohort. This means that suicide mortality in the young birth cohort has had more significant effects on total mortality, and indicates that the age-specific reasons behind this increasing suicide rate in South Korea warrant further investigation.

### Age- and gender-specific differentials in determinants of suicide

Risk factors for suicide seem to differ by age and gender. A recent British study [16] showed that the suicide rates for males under age 45 almost doubled between 1950 and 1998, while there were declining trends for older males and for females. The authors argued that the unfavourable trend in the younger male group was related to social disintegration, such as unemployment, divorce, alcohol and drug abuse, decline in marriage, and income inequality. On the other hand, a decrease in suicide rates of older people in England and Wales was attributed to a social safety network, namely the extended National Health Service health provision and wide-spread use of antidepressant agents as a result of an increased gross domestic product. In addition, the follow-up study between 1998 and 2005 in England and Wales reported a declined suicide rate among younger people through the reduction of major risk factors, such as unemployment [17]. Our study showed a pattern in age-specific suicide rates that differed from the UK study. Although an increasing trend in suicide rates was observed in both young and elderly people, age-differential effects on suicide were displayed in opposite directions, in terms of absolute and proportional suicide mortality comparison. The increase in the absolute suicide rates was much greater in older people, in contrast to the proportional rates, which were greater among the younger group aged under 45. A recent UK study [18] suggested that the increase in the younger group should further deteriorate potential years of life lost (PYLL) due to suicide in the country. Our results suggest that adolescent/adult suicide in Korea cannot be masked behind the absolute value of the elderly suicide rates in total mortality. In particular, younger females showed the steepest increase in later periods, calling for further studies on the social phenomena behind these trends.

### Recent rise in suicide mortality in South Korea and possible explanations

As mentioned, the Asian economic downturn in 1997 evoked an increase in suicide rates with a ripple effect occurring even after economic recovery in Korea. The sudden increase of suicide mortality for the short term before and after 1997/98 concords with previous studies [15,19]. Economic recession or crisis showed different effects on the suicide rate or health status by country [20]. Thailand was the country most affected by economic downturn like South Korea in 1997/98. The suicide rate in Thailand surged in 1999 and declined after recovery of the crisis (5.9 in 1977, 8.6 in 1999 and 7.8 in 2002)[21]. Russia is another typical example illustrating the effect of economic crisis on suicide rates. Suicide rose just after the currency crisis in 1998 and it fell with the recovery of economy [22]. However Finland which experienced a higher rising unemployment rate with the deep economic recession during 1990s did not show the increase of suicide rate [23]. Thus, it would be related to the differences in social networks between countries [16].

We will now explore the possible reasons underlying the recent increasing trends of suicide rates. First, we can examine the conflicting social conditions for the Korean elderly in private and public care. As longevity increased substantially (from age 65.1 to 75.1 in men and from age 73.4 to 81.9 in women, 1986–2005) [24], preparation for later life has become more critical for the elderly of more recent cohorts. Since the 1980s, the Korean government has actively initiated diverse programs for the elderly under the newly established Law of Elderly Welfare, such as old age pensions and home care [25]. Yet, social benefits and social safety networks provided by the government were insufficient, while family support to elderly parents seemed to weaken [26]. Some have argued that the Confucian ideology of *Hyo *(filial piety) has substantially weakened in South Korea. '*Hyo*' is a concept of blind love and respect to one's parents. Traditionally in the Korean culture, mostly sons had a responsibility of taking good care of parents in their elderly stages of life [27]. Compared to other OECD countries, labour market participation by the elderly aged 65+ was much higher in Korea (30% versus 10% in Sweden and 15% in the USA) [28]. Therefore, the quality of life for elderly people may be worse after retirement, and some are required to work for a living in their oldest age. This may have caused the increase in elderly suicide, which differs from Western statistics, despite the GDP increase [16]. Second, income inequality in Korea has increased significantly following the recent economic downturn. According to the Gini index as a measure of income inequality, the inequality for urban workers remained quite stable up to 1997, but increased sharply after 1998 (0.283 in 1997 to 0.320 in 1999) [29]. Labour market reconstruction after the 1997 economic crisis also affected the increase in unemployment rates and part time workers, with further effects on younger people. As in the UK, changes in the labour market and job insecurity most likely contribute to the rise in suicide rates among younger people in Korea. Third, social integration in Korean society seems to have become weaker in the recent decade. We can find related evidence in diverse social indicators. The divorce rate has increased (crude divorce rate was 1.4 per 1000 in 1994, 2.5 in 1998 and 2.6 in 2005 [11] and the phenomenon of a decline in the number of marriages as well as increased late marriage was further accelerated (crude marriage rate went from 9.3 per 1000 in 1990 to 6.5 in 2005)[30]. Moreover, the age of Koreans for first marriage increased from 27.8 in men and 24.8 in women in 1990 to 30.9 in men and 27.7 in women in 2005 [31]. As a result, total fertility rate has decreased from 1.60 per woman in 1986 to 1.08 in 2005[32].

Interestingly, the increase in suicide rates was more evident in women than in men. Weakening social integration may have affected more women than men. This can be supported by a study that showed that women with children were less likely to commit suicide [33]. Finally, technology seemed to affect the recent suicide rate increase, including Internet or mass media [34]. Wide use of the internet in Korea (the broadband infrastructure is the world's state-of-the-art and web users reached almost 80% in 2005 [12]) may be an important cause of the increase in suicide deaths, particularly among the younger birth cohort. The Internet can be the means to exchange tips about suicide and to bring together people having suicidal thoughts. The pressure to succeed at school and work may be so intensive that young Koreans are more willing to end their lives. As a counteraction, the Korean government now operates a watchdog in cyberspace, ordering the shutdown of 566 blogs, chat groups, or web postings that encouraged suicide in 2005 [35].

### Limitations

There are some limitations to the interpretation of suicide data, and some intrinsic properties of suicide in terms of "suicide definition" and "misclassification error" cannot be avoided. The meaning of suicide may differ by location or culture, which sometimes leads to invalid information particularly in comparative studies. Recently, for the analysis of suicide trends, it is recommended that the undertermined death (e.g. ICD-10 Y10–Y34) cases need to be considered as part of the suicide mortality to avoid the misclassification [36]. However the data on undetermined death rate was not available for the whole study periods, but available only since 2001. The level of suicide death rate might not be significantly affected by the undetermined death, because it was similarly on the steady increase like that of the suicide rate (the undetermined death rate (ICD-10 Y10-4) per 100,000 from 4.6 to 6.7 in male and from 2 to 3.2 in female from 2001 to 2005). In addition, the level of exclusiveness of the investigation (*i.e*., autopsy) or the social stigma of suicide may affect reported suicide rates in a given society [37].

Korean data on suicide are dependent mostly on two resources. One is cause-specific death statistics based on the certificate of death issued by the National Statistical Office (NSO) and the other is death statistics based on thorough investigations of death cases by the National Prosecution Office (NPO). These two data sources supply different information. Since 2002, NSO's death statistics have begun to include background information on the NPO's report, which may have affected the abrupt increase in suicide rates in the 2002 Korean statistics. However, our analysis suggested that suicide rates have been increasing continuously from 1996 through 2005.

In the current study, we did not attempt to analyze the relationships between macro-social indicators and annual suicide rates. This is because the increase in suicide rates corresponded not only with short term drop in per capita gross national income; but also with a long term increase in per capita gross national income. A simple analysis would obscure these two effects with opposite direction. These conflicting patterns suggest that these social indicators would be only markers of certain social changes, and the true social determinants are not measured properly. Furthermore, different age groups might respond to the social changes in different ways, possibly because of different social risk factors specific to the age group. Our study points to the difficulty in identifying social determinants of suicide with ecological analysis. Future studies should include the analysis of diverse social risk factors relevant to different age groups. In addition, individual level information on the exposure to such social risk factors may need to be used in combination with macro-social indicators.

## Conclusion

Our analysis suggests that both absolute and proportional increases should be considered in understanding suicide epidemiology according to age and gender. There were distinct cohort effects underlying the increasing suicide rates in Korea, particularly among younger age groups. Social changes appeared to have increased suicide risk factors differentially according to age. The development of suicide prevention strategies and assessment of their effectiveness should be considered separately for the young and elderly populations in South Korea.

## Competing interests

The authors declare that they have no competing interests.

## Authors' contributions

JWK undertook data extraction and analysis and wrote the first draft. HRC wrote the revised version. SIC formulated research questions, designed the analysis, and contributed to writing the drafts. All authors interpreted the results and contributed to the final draft.

## Pre-publication history

The pre-publication history for this paper can be accessed here:


